# Preliminary Education, Etc.

**Published:** 1859-02

**Authors:** 


### PRELIMINARY EDUCATION, ETC.

In a recent number of the *Peninsular and Independent*, we are accused, as usual, of having misrepresented the Medical Department of the University of Michigan, when we stated, not long since, that the faculty of that school had taken especial pains to make the profession generally believe they had actually complied with the recommendation of the American Medical Association, in relation to exacting a certain standard of preliminary education for their students, while in truth they had done nothing of the kind. If our readers can turn to almost any of the annual announcements of the Michigan University between 1850 and 1856, they will find not only a general claim of having complied with the recommendations of the American Medical Association, but also a verbatim copy of the identical standard of preliminary qualifications adopted by the last-named Association at its organization, in 1847, set forth as the standard of their school. True, the circulars did not say how or when this standard was enforced—whether on *admission* to the school or some other time. But the allusion to the recommendations of the Association, and the copying of the language of one of these recommendations, was well calculated to make the reader believe that they had actually adopted it. And not only so, but Dr. Z. Pitcher, of Detroit, an Emeritus Professor, and, we believe, President of the Medical Department of that University, in his Report on Medical Education made to the American Medical Association in May, 1853, used the following explicit language:

"By the medical faculty of the University, all candidates for the degree of M.D. *are rigidly required to comply with the conditions of the National Medical Association*, except attendance upon hospital cliniques."

Here we are told, not in a college circular, nor in a partisan medical journal, but on the floor of the great American Medical Association, and in the presence of five hundred delegates, representing nearly all the States of the Union, that "all candidates, etc., are *rigidly* required to comply with the conditions of the National Medical Association," with the single exception named. The exact "*conditions*" recommended by the National Association on the subject of preliminary education, we quoted in an editorial in the last (January) number of *Journal*. Let the reader compare the resolutions there quoted from the transactions of the Association with the statement here quoted from the report of Dr. Pitcher, and ask himself whether the inference is not plain and positive, that the University of Michigan required a standard of preliminary education for the admission of those who intended to become candidates for graduation? Would any live man have imagined that the "*rigidly required to comply*," etc., only meant that the candidates were required, during the *second* course of lectures, to write six or seven essays on medical subjects, instead of one, as in other colleges? We do not believe Dr. Pitcher meant to deceive by his statement. On the contrary, we believe he expected a standard of preliminary requirements was to be actually exacted; and that he has since used his influence honestly to have the University carry into practical effect all that he represented in his report. But he has been wholly overruled by the active members of the Medical Faculty, and all the pretensions on this subject, according to Dr. Palmer's own showing, have had no other practical reality than the exaction of several short essays from the candidates for graduation during the last course of lectures. We have called the attention of our readers to this subject, both in the present and preceding numbers of the *Journal*, not from any desire to criticise unnecessarily the doings of our neighbors in the Peninsular State, but for the purpose of directing the attention of our readers to the general subject of preliminary education for medical men, and the true nature of the recommendations of the National Association on the subject.

It will be seen by the resolutions quoted in the January number, that individual members of the profession, in their capacity of private preceptors, are expected to take the first and most important step in relation to this matter. It is by the private preceptor, and not the college, that students are actually introduced into the profession. And hence, whatever preliminary education is necessary to qualify a young man to enter upon strictly medical studies with propriety, should certainly be exacted when he first makes application for admission into the practitioner's office as a student of medicine. And this is precisely what the National Association has recommended, repeatedly, and with much unanimity.

We hope the members of the Illinois State Medical Society will reflect on this subject, and be prepared to take definite action on it at the annual meeting on the first Tuesday in June next. If the State Society will take such action as will require all its members to ascertain, and certify to, the preliminary education of all who may apply for admission into their offices as students, the same thing will speedily be done by all the more local societies; and thus a firm and practical foundation will be laid for the most important improvement in the character of the profession of which it is susceptible.

